# A Review of the Relationship between Gel Polymer Electrolytes and Solid Electrolyte Interfaces in Lithium Metal Batteries

**DOI:** 10.3390/nano13111789

**Published:** 2023-06-01

**Authors:** Xiaoqi Yu, Zipeng Jiang, Renlu Yuan, Huaihe Song

**Affiliations:** State Key Laboratory of Chemical Resource Engineering, Beijing Key Laboratory of Electrochemical Process and Technology for Materials, Beijing University of Chemical Technology, Beijing 100029, China; yxqyx02060304@163.com (X.Y.); zipengjiang@163.com (Z.J.); rlyuan2016@163.com (R.Y.)

**Keywords:** lithium dendrite, gel polymer electrolyte, solid electrolyte interface, lithium metal battery

## Abstract

Lithium metal batteries (LMBs) are a dazzling star in electrochemical energy storage thanks to their high energy density and low redox potential. However, LMBs have a deadly lithium dendrite problem. Among the various methods for inhibiting lithium dendrites, gel polymer electrolytes (GPEs) possess the advantages of good interfacial compatibility, similar ionic conductivity to liquid electrolytes, and better interfacial tension. In recent years, there have been many reviews of GPEs, but few papers discussed the relationship between GPEs and solid electrolyte interfaces (SEIs). In this review, the mechanisms and advantages of GPEs in inhibiting lithium dendrites are first reviewed. Then, the relationship between GPEs and SEIs is examined. In addition, the effects of GPE preparation methods, plasticizer selections, polymer substrates, and additives on the SEI layer are summarized. Finally, the challenges of using GPEs and SEIs in dendrite suppression are listed and a perspective on GPEs and SEIs is considered.

## 1. Introduction

Against the background of the carbon peak and carbon neutrality era, large-scale development of clean energy (wind energy, solar energy, etc.) is imminent. However, clean energy has the problem of an uneven distribution of time and space. Therefore, research on safe and efficient energy storage systems is extremely urgent [1]. Current commercial batteries (lead-acid batteries, nickel-cadmium batteries, LIBs, etc.) cannot meet the increasing energy storage requirements due to their low energy density [2]. Metallic lithium (Li) possesses a theoretical capacity of 3860 mAh/g and a low reduction potential (−3.04 V vs. a standard hydrogen electrode) [3,4]. The energy density of lithium metal batteries (LMB) is 3~5 times than that of lithium ion batteries [5], which has attracted extensive attention.

In general, Li ions are reduced to Li^0^ during the charging process. Li^0^ is oxidized to Li^+^ during the discharge process [6]. However, during the cycle process, Li^0^ is unevenly deposited on the surface of the anode, forming various lithium dendrites due to the thermodynamic instability of lithium metal in the organic liquid electrolyte [7]. Uncontrolled dendrite growth may penetrate the separator, causing a series of safety problems such as internal short circuits, overheating, and explosions. Moreover, reparation of the fragile solid electrolyte interface (SEI) films happens at the expense of consuming the electrolyte, resulting in low Coulombic efficiency [8,9]. Therefore, the formation of a robust SEI layer and uniform lithium deposits are essential to LMB stability [10,11].

At present, the research on SEI layers and the even deposition of lithium examines electrolytes and lithium metal anode (LMA) in situ protective layers (customizable artificial SEI layers) [12,13,14]. However, the artificial SEI cannot be repaired by the reduction products of electrolytes when it becomes damaged during the course of cycling [15]. Liquid electrolyte (LE) additive regulates SEI components to promote lithium deposition. Unfortunately, the finite service life of the additive is not good for improving long-term cycles [16,17]. Moreover, polymer electrolytes (PEs) have attracted attention as a substitute for LE and separators. PE are divided into solid polymer electrolytes (SPEs) and gel polymer electrolytes (GPEs) [18,19,20]. However, SPEs have the disadvantages of poor room-temperature (RT) ionic conductivity and high interfacial resistance [21,22,23]. Fortunately, as a combination of SPEs and LEs, GPEs can effectively improve RT ionic conductivity and decrease interface resistance [24,25]. Furthermore, the functional groups of GPE polymers can anchor lithium salt anions, which prevents the formation of space charge zones at the lithium anode interface. It also reduces the occurrence of side reactions caused by anions. This facilitates the formation of a uniform and robust SEI layer during cycling [4].

Recently, there have been few reviews on the relationship between GPEs and SEIs. This review first introduces the mechanism of GPE-inhibiting lithium dendrites. Meanwhile, the importance of SEIs formed using GPEs in dendrite suppression is suggested. Secondly, the advantages of GPEs forming SEIs are discussed. Then, the effects of GPE preparation methods, additives, plasticizers, and polymer substrate selections on SEIs are described. Finally, the relationship between GPEs and SEIs is summarized and the future development of GPEs and SEIs is considered.

## 2. GPE Builds the Dendrite-Free Lithium Metal Anode

### 2.1. The Development of GPE

The research and development of solid polymer electrolytes (SPEs) began with Wright’s discovery of ionic conductivity in the PEO-alkali metal ion complex in 1975. He provided a new idea to solve the problem of LE leakage in lithium batteries [22]. However, SPEs have the defects of low RT ionic conductivity and high interfacial resistance. In this context, gel polymer electrolytes (GPEs), the intermediate products of SPEs and liquid electrolytes (LEs), have the advantages of both electrolyte forms. GPEs have the advantages of the good machining performance of polymer electrolytes and the high RT ionic conductivity of LEs, while also improving the energy density of lithium batteries by replacing separators [26,27,28]. Moreover, GPEs are mainly divided into two categories: physical or chemical cross-linking GPEs. In 1975, Feuillade et al. [29] prepared a physically cross-linked GPE by utilizing polyvinyl acetals (PVAs) and polyacrylonitrile (PAN); additionally, they prepared a chemically cross-linked GPE using hydroxylated polyvinyl acetals and poly(vinylidene fluoride-hexafluoropropylene) (PVDF-HFP). The electrochemical and intrinsic test results showed that the chemically cross-linked GPE had decent conductivity, mechanical properties, and swell ability. In 1983, Iijima et al. [30] used polymethyl methacrylate (PMMA) as a gelatinizing agent to prepare GPEs, the RT ionic conductivity of which reaches up to 1~3 mS·cm^−1^. In addition, there was no leakage phenomenon observed, even after being stored at 60 °C for a month. Furthermore, following Bellcore’s development in 1994, GPEs entered a period of rapid development in LIBs.

**Figure 1:**
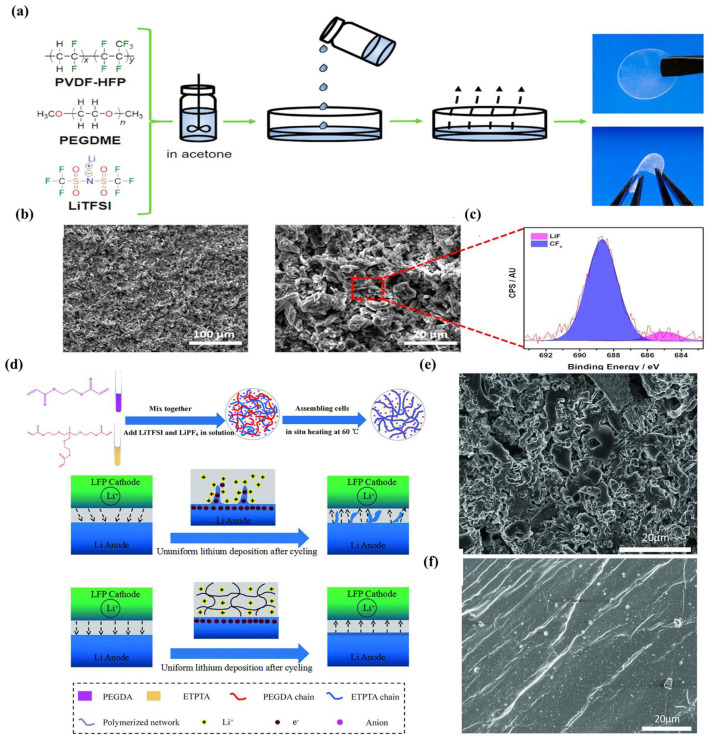
(**a**) Schematic illustration of a GPE; (**b**) SEM images of a Li anode; (**c**) an F 1s region corresponding to the XPS spectra of Li^0^ deposition [43]. Reproduced from Ref. [43] with permission from American Chemical Society; (**d**) a step process for in situ polymerization of GPEs; (**e**) the Li anodes of the LiFePO_4_||separator liquid electrolyte||Li cell and (**f**) LiFePO_4_||GPE||Li cell [44]. Reproduced from Ref. [44] with permission from Wiley.

GPE composition has its own role [31]. As the sources of charge carriers, lithium salts require low dissociation energy to promote the movement of lithium ions. Commonly used lithium salts include LiPF_6_, lithium bis(trifluoromethanesulphonyl)imide (LiTFSI), lithium bis(fluorosulfonyl)imide (LiFSI), LiNO_3_, LiClO_4_, and LiBF_4_, etc. [23,32,33]. The plasticizers are linear carbonate (EMC, DMC, and DEC, etc.), cyclic carbonate (PC, EC), and ether (DME, TEGDME, DOL, THF) [34,35,36,37]. The carbonate plasticizer is conducive to the dissolution of lithium salt. Compared to a carbonate liquid electrolyte, an ether liquid electrolyte has the advantages of lower voltage lag and higher Coulombic efficiency. However, ether liquid electrolytes have the drawback of volatilization [38]. The polymer substrate can facilitate the dissociation of lithium salts and provide excellent mechanical properties. In addition, LE is fixed in the polymer substrate crosslinking network to reduce the contact area between the LE and the anode. Common polymer substrates include polyvinyl alcohol (PVA), polyethylene glycol (PEG), polyacrylonitrile (PAN), polyethylene oxide (PEO), polyvinylidene fluoride (PVDF), polymethyl methacrylate (PMMA), etc. [39,40,41,42]. Castillo et al. [43] introduced polyethylene glycol dimethyl ether (PEGDME) and LiTFSI into PVDF-HFP to prepare GPE (Figure 1a). As luck would have it, GPEs produce tight LiF-rich SEIs upon contact with LMAs. No obvious lithium dendrite structures can be seen in the SEM images (Figure 1b,c). Moreover, GPEs contain dilithium salts that favor the dissociation of lithium salts and reactions in the positive direction. Fan et al. [44] prepared a novel dilithium salt GPE through in situ polymerization introducing LiTFSI-LiPF_6_ into a 3D cross-linked network. The irregular deposition of lithium in LEs leads to the appearance of a large number of dendrites. In contrast, the lithium ions’ uniform distribution in GPEs is conducive to lithium deposition, thus avoiding the formation of dendrites (Figure 1d). Moreover, the stable SEIs formed between GPEs and LMAs effectively inhibit the growth of lithium dendrites (Figure 1e,f).

### 2.2. Effect of GPE Composition on Lithium Ionic Conductivity

#### 2.2.1. Lithium Salts

**Figure 2:**
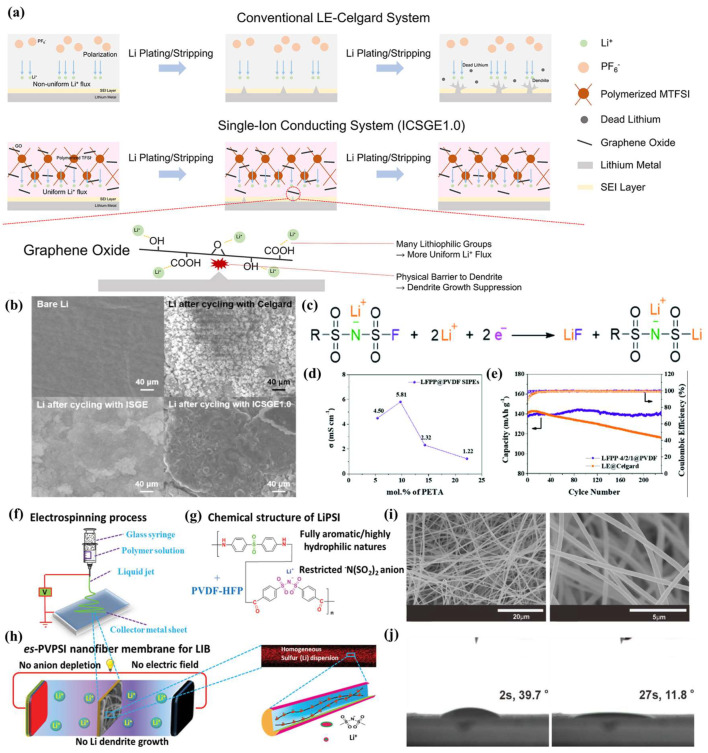
(**a**) Graphical illustration of lithium dendrite growth suppression by ICSGE1.0; (**b**) surface SEM images of lithium metal before cycling and cells with LE-Celgard, ISGE, ICSGE1.0, respectively, after cycling [48]. Reproduced from Ref. [48] with permission from Elsevier; (**c**) the SEI formation mechanism of the LFPP@PVDF SIPE on the lithium anode; (**d**) ionic conductivity of LFPP@PVDF SIPEs with various ratio fractions at RT; (**e**) cycle performance of Li|LFPP-4/2/1@PVDF SIPE|LFP batteries at 0.2 C [49]. Reproduced from Ref. [49] with permission from Royal Society of Chemistry; schematic illustration of the fabrication (**f**), composition (**g**), and operation (**h**) of the *es*-PVPSI nanofiber-based membrane, acting as single-ion conducting polymer electrolyte in LIBs; (**i**) SEM images of the *es*-PVPSI nanofiber membrane; (**j**) the solvent contact angle (EC/DMC (v:v = 1:1)) evolution on the *es*-PVPSI nanofiber membrane at 2 s and 27 s [50]. Reproduced from Ref. [50] with permission from Wiley.

The low migration volume of lithium ions leads to serious concentration polarization, which affects the uniform deposition of Li^0^ [45]. According to the results of previous theoretical calculations, the Sand’s time of lithium dendrite formation is proportional to the number of lithium ion transfers. In the case of lithium ion transfer alone, the Sand’s time is extended indefinitely to create a dendrite-free anode [24,46,47]. Notably, lithium salt anions are anchored by functional groups on their polymer skeleton, which facilitate lithium ion transfers. Jeong et al. [48] prepared cross-linked single-ion conducting gel polymer electrolytes (CSGE#s) through in situ polymerization using methacrylate graphene oxide (MGO), lithium 1-[3-(methacryloyloxy)propylsulfonyl]-1-(trifluoromethane sulfonyl)imide (LiMTFSI), and PVDF-HPF (Figure 2a). Compared with Celgard with an ordinary LE, CSGE1.0 can inhibit the formation of lithium dendrites well (Figure 2b). Zhong et al. [49] prepared a LiSFSI–PETMP–PET4A@PVDF single ion polymer electrolyte (LFPP@PVDF SIPE) using lithium [(4-styrenesulfonyl)(fluorosulfonyl) imide] (LiSFSI), pentaerythritol tetrakis (3-mercaptopropionate) (PETMP), and pentaerythritol tetraacrylate (PET4A) with in situ photopolymerization. As a single ion conductor, the SO_2_-F group of LiSFSI is conducive to the generation of an SEI on the anode surface (Figure 2c). LFPP@PVDF SIPE has a high ionic conductivity of 5.81 mS·cm^−1^ and a lithium ion transfer number of 0.91. Moreover, the LiFePO_4_ || LFPP @ PVDF SIPE || Li cell initial capacity is 140 mAh·g^−1^. There is no significant capacity attenuation after 230 cycles at 0.2 C (Figure 2d,e). Li et al. [50] prepared porous nanofiber single-ion conducting polymer membranes (*es*-PVPSI) using PVDF-HFP and lithium poly(4,4′-diaminodiphenylsulfone, bis(4-carbonyl benzene sulfonyl)imide) (LiPSI) and electrospinning (Figure 2f–h). The *es*-PVPSI has a porous structure, and it can be seen from the contact angle test that the absorption rate of LEs (1M LiPF_6_, EC:DEC = 1:1, *v*/*v*) is fast, while the adsorption uptake ratio (144.57%) is twice as high as that of polypropylene (PP) separators (Figure 2i,j). The *es*-PVPSI lithium ion transfer number is 0.85.

In general, lithium salts with low lattice energy have excellent stability, solubility, and high conductivity [51,52]. Subadevi et al. [53] studied the effects of different lithium salts (LiClO_4_, LiBF_4_, LiCF_3_SO_3_) on the electrochemical performance of GPEs using PVDF-PEMA as a polymer substrate and EC and PC as plasticizers. Experimental results showed that GPEs containing LiClO_4_ had the highest ionic conductivity, because LiClO_4_ is more easily dissociated and its anion size is larger than those of LiBF_4_ and LiCF_3_SO_3_. Notably, LiClO_4_ and LiBF_4_ have low ion pairing, which is favorable for ionic conductivity. Lithium ion transport occurs mainly in the amorphous region of the polymer. In addition, a large number of polar groups on the polymer surface coordinate with lithium ions to improve ionic conductivity. Liu et al. [54] prepared a PEGDA/CA GPE by introducing cellulose acetate (CA) into PEGDA. The PEGDA/CA GPE was activated in a LE (1 M LiPF_6_ in EC: DEC = 1:1 *v*/*v*). CA containing a large number of ether bonds and carbonyl groups enhanced ion-dipole interactions that weaken ion pairing, which promoted Li^+^ transference.

Concentration polarization is avoided when the lithium ion migration number of GPEs is close to 1 [55,56]. However, there is a sufficient but unnecessary relationship between the increase in the lithium ion migration number and the increase in ionic conductivity [57,58]. Therefore, the improvement of the GPE ionic conductivity is the result of the synergistic effect of a high LE absorption rate, the use of a polymer substrate as a cationic conductor, and a stable SEI.

#### 2.2.2. Polymer Substrates

**Figure 3:**
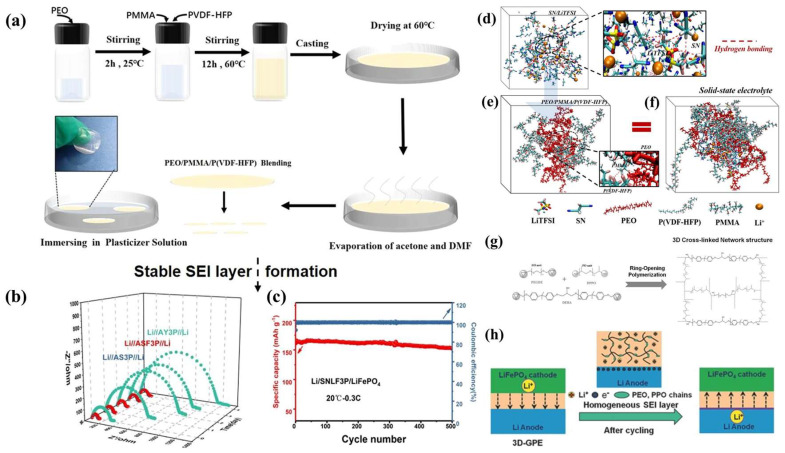
(**a**) Synthetic scheme of PEO/PMMA/P(VDF-HFP) GPEs; (**b**) the time evolution of the impedance response of Li/GPEs/Li symmetrical batteries at RT; (**c**) cycling performances of the Li/SNLF3P/LiFePO_4_ battery at 0.3 C and the MD−calculated structure of a different system; (**d**) SN/LiTFSI; (**e**) PEO/PMMA/PVDF-HFP; (**f**) SN/LiTFSI combined with a PEO/PMMA/PVDF−HFP system [62]. Reproduced from Ref. [62] with permission from Elsevier; (**g**) schematic of the synthesis of the GPE membrane; (**h**) 3D−GPE during the Li plating/stripping [63]. Reproduced from Ref. [63] with permission from Wiley.

The carbonate liquid electrolyte has a high reactive activity with the LMA, meaning it easily generates lithium dendrites. The LE was fixed in the GPE polymer substrate to reduce the contact area between the LE and the LMA. This can alleviate the problem of lithium dendrite growth [59]. The polymer substrate of GPEs should have the following properties: (1) efficient chain segment movement to promote the migration of lithium ions; (2) facilitated dissociation of special atoms or groups from lithium salt; (3) a low Tg value, which corresponds to more amorphous regions in the polymer substrate, which are conducive to improving ionic conductivity; (4) good thermal stability; and (5) excellent electrochemical performance [46,60,61]. Ye et al. [62] used PMMA, PVDF-HFP, PEO, and LE succinonitrile (SN)/LiTFSI/FEC to prepare 3D-GPE (Figure 3a).The introduction of plasticizer FEC into the polymer matrix can reduce the interface resistance and promote the formation of a stable SEI (Figure 3b,c). Moreover, the molecular dynamic (MD) simulations indicate that lithium ions interact strongly with the ether group of PEO and the cyanide group of SN, which promote the dissociation of LiTFSI (Figure 3d–f). Lu et al. [63] used diglycidyl ether of bisphenol-A (DEBA), poly(ethylene glycol) diglycidyl ether (PEGDE), and diamino-poly(propylene oxide) (DPPO) to prepare 3D-GPE through the ring-opening polymerization reaction (Figure 3g). EO and PO groups have a high affinity for the LE (1M LiPF_6_, EC/DMC = 1:1, *v*/*v*), which firmly wraps the solvent molecules in the polymer network. Moreover, 3D-GPE forms a highly uniform SEI layer on the lithium electrode. The SEI layer and the GPE dense network structure act synergically to inhibit dendrite growth (Figure 3h).

**Figure 4:**
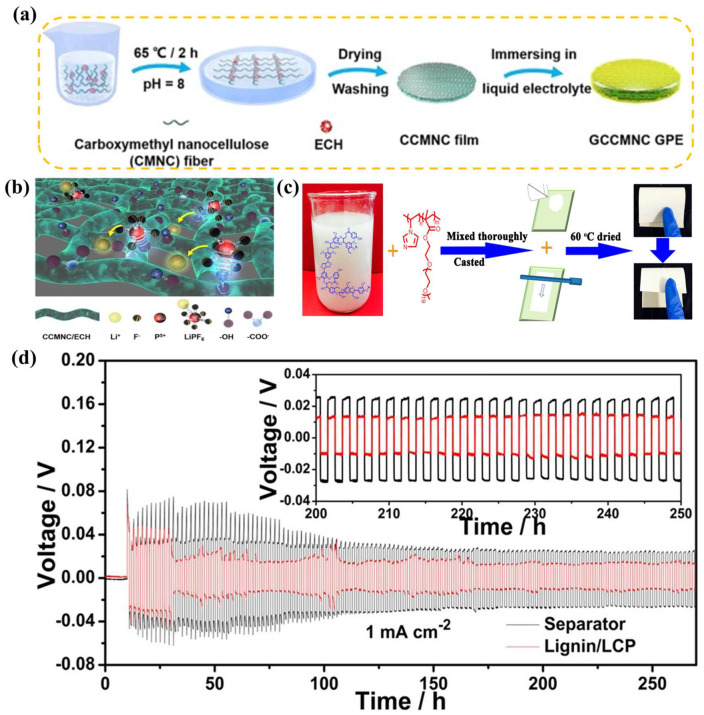
(**a**) schematic illustration of the preparation process of CCMNC film and GCCMNC GPE; (**b**) schematic illustration of the mechanism of lithium ion transportation in GCCMNC GPE [68]. Reproduced from Ref. [68] with permission from Elsevier; (**c**) preparation route of the lignin/LCP membrane; (**d**) galvanostatic cycles for Li||separator−liquid electrolyte||Li and Li||lignin−based electrolyte||Li symmetrical cells under a current density of 1 mA cm^−2^ [69]. Reproduced from Ref. [69] with permission from American Chemical Society.

In the initiative of green chemistry, eco-friendly and low-cost natural polymer materials, such as cellulose, protein, and sodium alginate, etc., have attracted attention [42]. Moreover, the high flexibility and elastic modulus of natural polymer materials provide good mechanical properties for GPEs to tolerate anode volume changes. Surprisingly, the polar groups (-NH_2_, -OH, -C=O, etc.) and heteroatoms (N, O, S, etc.) present in natural polymer materials effectively anchor anions using hydrogen bond interactions [64,65,66]. Wen et al. [67] prepared a 3D porous GPE (LA-PEO-PAM-3-1-1) using natural polymer alginate granules (LA), polyacrylamide (PAM), and PEO. LAs not only provide additional lithium ions, but also promote the dissociation of LiTFSI. Meanwhile, the mechanical and electrochemical properties of the GPE are improved due to the strong hydrogen bond interaction between PAM and the LA. Wang et al. [68] used carboxylated nanocellulose (CMNC) with anionic properties and epichlorohydrin (ECH) to synthesize an environmentally friendly GPE (CCMNC) (Figure 4a). A large number of hydroxyl groups in CMNC enhance the mechanical properties of GPEs through intermolecular or intramolecular hydrogen bonding. Moreover, CCMNC’s strong hygroscopic and porous structure immobilizes LEs (1 M LiPF_6_, EC/DMC/DEC = 1:1:1, W/W/W) in the polymer network, alleviating dendrite problems caused by LEs’ high reactivity with lithium anode (Figure 4b). Wang et al. [69] prepared lignin-based films using lignin and linear binder poly(N-vinylimidazole)-co-poly(poly(ethylene glycol) methyl ether methacrylate) copolymer (LCP) (Figure 4c). The film was activated in a LE (TC-E201), had an absorption rate of 276%, and reached swelling saturation at 16 S. Moreover, the lignin base electrolyte can quickly generate a stable SEI after contact with lithium metal electrodes. Therefore, Li|| lignin-base electrolyte ||Li cells generate less potential time (~30 h) than Li|| Celgard 2300-LE ||Li cells (~110 h) (Figure 4d).

To sum up the mechanical properties of GPEs, LE absorption rate and ionic conductivity are related to polymer structure, functional group properties, and heteroatomic types. The ether group is a two-ionic conductor which dissociates lithium salts and anchors anions to promote lithium ion transference. Meanwhile, the heteroatoms (N, S, P, etc.) are indispensable components of polymer substrates. The complex reaction between heteroatoms and lithium ions is conducive to lithium ion transference. Moreover, the uniform structure with an appropriate pore size improves the LE absorption rate and improves the poor mechanical properties of the polymer substrates after activation in a LE. However, in this case, too much is too little—a higher LE absorption is not always better. This is mainly because an excessive amount of LE in a GPE can cause security problems (liquid leakage, combustion, or even explosion), which is the opposite of what LEs are designed for.

#### 2.2.3. GPE Additives

**Figure 5:**
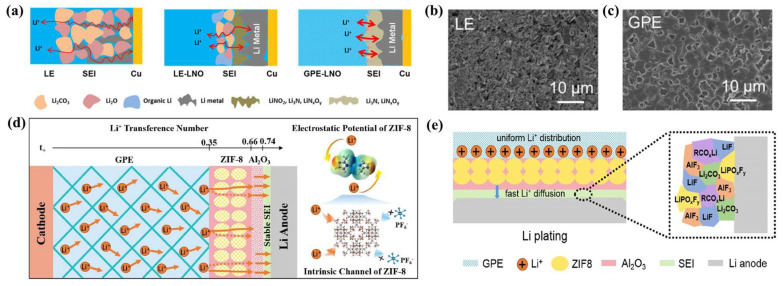
(**a**) Schematic representation of the plating and stripping process in LE, LE-LNO, and GPE-LNO electrolytes based on the operando NDP and XPS results; (**b**) SEM measurement at 0.2 mA/cm^−2^ in the LE and (**c**) at 0.2 mA/cm^−2^ in the GPE [75]. Reproduced from Ref. [75] with permission from American Chemical Society; (**d**) schematic diagram of the heterostructured GPE, the increase in the lithium ion transference number with the varying structure, and the electrostatic potential distribution and intrinsic channel of ZIF-8, (**e**) schematic illustrations of SEI components formed on the interface between GPE-ZIF8-Al_2_O_3_ film and the Li anode [77]. Reproduced from Ref. [77] with permission from American Chemical Society.

GPE additives have a great impact on dendrite inhibition. Qualified additives should have high mechanical properties, high chemical/electrochemical stability, easy dispersion, and should facilitate lithium ion transport. Commonly used additives are SiO_2_, BN, LiNO_3_, MOF, FEC, and ceramic nanoparticles [70,71,72,73,74]. For example, the addition of LiNO_3_ to ether or carbonate-based liquid electrolytes, even in small amounts, can significantly improve the interface chemical formation of a Li_3_N-rich SEI. Liu et al. [75] prepared a carbonate-based GPE with LiNO_3_ as an additive, and detected lithium metal deposition/stripping using an operando neutron depth profile (NDP). Nitrate ions can alter the nucleation of lithium metal, leading to spherical metal nucleation and growth to form a densely structured SEI and inhibit the formation of lithium dendrites (Figure 5a). Furthermore, the high conductivity of Li_3_N reduces the overpotential of the lithium anode. Compared to the bilayer SEI formed by adding LiNO_3_ to a LE, the combination of LiNO_3_ and a polymer substrate effectively generates a SEI that is thin, uniform, and LiNO_2_-free. This SEI significantly inhibits the generation of porous/dendritic lithium dendrites (Figure 5b,c). Shim et al. [76] introduced multilayer hexagonal boron nitride (BN) nanosheets functionalized with the multifunctional additive perfluoropolyether (PFPE) into PVDF-HFP to prepare G-CFBN. BN has a graphene-like structure. B-N bonds have ionic properties, which provide them with excellent electrical insulation, mechanical properties, and electrochemical stability. In addition, thanks to its Lewis acid properties, the N atom of BN interacts with the lithium salt anions in LE (1M LiTFSI, EC/DEC = 1:1, *v*/*v*) to promote lithium ion transfer. Therefore, Li || G-CFBN || Li can reach stable circulation 1940 h at 1 mA·cm^−2^, while LiFePO_4_ || G-CFBN || Li °C.

When ceramic particles are introduced into polymer electrolysis, the phenomenon of agglomeration and phase separation occurs. In addition, the excessive size and amount of filler will affect the ionic conductivity and energy density of the battery. In order to combine the advantages of ceramic fillers and polymer electrolytes to fully exploit their properties, it is necessary to establish a stable interface with a low diffusion barrier on the GPE surface. Cui et al. [77] introduced MOF and Al_2_O_3_ on one side of the polymer substrate to prepare a novel heterostructure GPE (Figure 5d). The ZIF-8 and (2-Methylimidazole zinc salt) Al_2_O_3_ coatings significantly improve the electrochemical performance of the GPE, which is mainly due to the homogenization effect of lithium ion transfer and the solvation effect of the two coatings. Al_2_O_3_ has a strong affinity with the LMA, which reduces nucleation overpotential and inhibits the generation of lithium dendrites. Moreover, Al_2_O_3_ interacts with LiPF_6_ to generate highly conductive AlF_3_. In addition, SEIs containing large volumes of AlF_3_ and LiF significantly promote charge transfer and reduce the diffusion barrier to stabilize the lithium anode/electrolyte interface (Figure 5e).

### 2.3. GPE Design

#### 2.3.1. Structural Design

**Figure 6:**
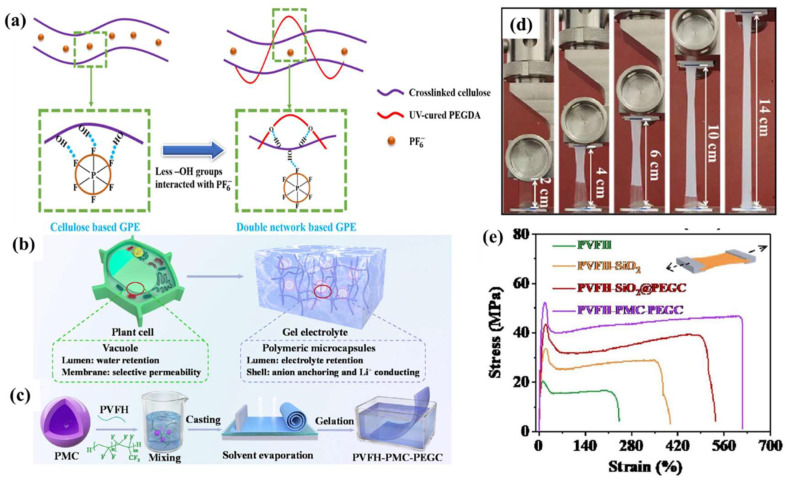
(**a**) a schematic to explain the interaction between anions and polymer chains [82]. Reproduced from Ref. [82] with permission from Elsevier; (**b**) a plant cell-inspired composite GPE; (**c**) the synthesis procedure of PVFH-PMC-PEGC; (**d**) optical image of the PVFH-TOC-PEG membrane during stress–strain measurement; (**e**) stress–strain curves of the PVFH-based membranes [83]. Reproduced from Ref. [83] with permission from Elsevier.

The mechanical properties of the polymer substrate decrease after absorbing the LE, then the lithium dendrites penetrate the GPE and destroy the SEI layer at the same time. The continuous repair of the SEI layer consumes a large amount of LE, leading to decreases in the Coulombic efficiency in the battery. Therefore, a tough GPE structure is essential [78,79,80]. Gou et al. [81] prepared different internal structures of GPEs by adjusting the degree of crosslinking of nanocellulose (NC). Moreover, the experiment found that the drying method for polymer substrates has a great impact on the mechanical properties of GPEs. Compared with forced air drying, freeze drying can eliminate the capillary effect between NC and effectively ensure the morphology and structure of polymer substrate. However, freeze drying reduces the mechanical properties of a polymer film. In addition, a dual-network structure can significantly improve the mechanical properties of the polymer substrate. The excessive crosslinking density leads to a decrease in the pore size, which is not conducive to the electrochemical performance of GPEs. Gou et al. [82] prepared a dual network structure GPE using cellulose and PEGDA through chemical crosslinking and UV radiation. The hydrogen bond formed by cellulose-OH and the PEGDA ether bond are beneficial to interfacial compatibility (Figure 6a). Interestingly, Zhai et al. [83] developed a bionic GPE (PVFH-PMC-PEGC) using PVDF-HFP and PMC-PEGC (Figure 6b,c). PMC acts as a lumen to promote the absorption of LE (1M LiTFSI, DME/DOL = 1:1, *v*/*v*). PVFH-PMC-PEGC acts as a cell membrane to conduct lithium ions and anchor anions. The vacuolar structure of the GPE enables the effective fixation of the LE, which leads the anode surface to be covered with a dense SEI layer. This is conducive to the uniform deposition of lithium. Moreover, the yield strength of PVFH-PMC-PEGC is up to 52.1 MPa, and the elongation at break is 615% (Figure 6d,e).

In addition, the thermostable, electrochemical stable glass fiber, or Celgard, introduced to the GPE can effectively solve the problem of mechanical property degradation after GPE activation [84,85,86]. Wu et al. [87] prepared a gel polymer electrolyte (GF-PBA) using poly(butyl acrylate), a glass fiber membrane, and LiTFSI. The introduction of glass fiber not only improves the mechanical properties of the GPE, but also promotes the dissociation of lithium salt by Si-O bonds. Moreover, Chen et al. [88] added poly(methyl methacrylate-acrylonitrile-butyl acrylate) [P(MMA-AN-BA)] solution to both sides of the PE Celgard and the prepared GPE membrane using the phase transformation method. The experimental results show that the mechanical properties of GPEs can be significantly improved by using PE Celgard as a support material, resulting in a fracture strength of up to 82.3 MPa.

#### 2.3.2. Functional Design

**Figure 7:**
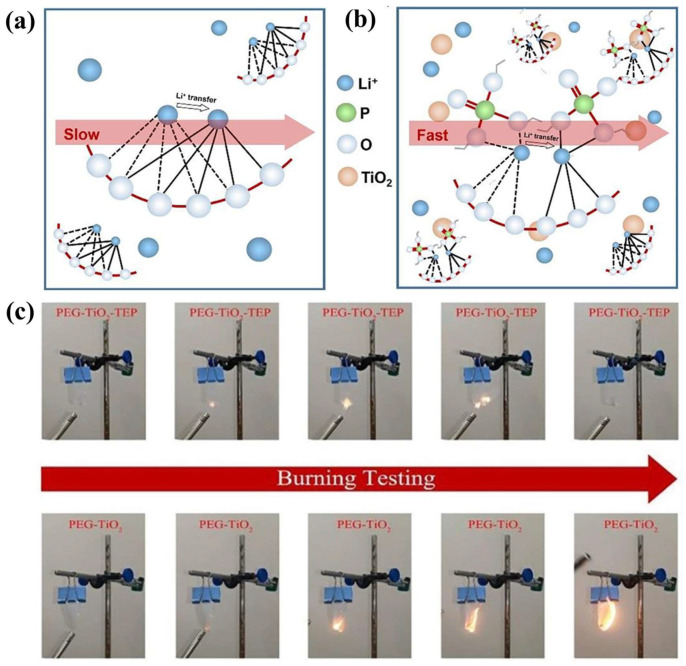
(**a**,**b**) Li ion conduction pathways in PEG PEs and PEG-TEP-TiO_2_ GPEs; (**c**) fire-resistant tests of the PEG and PEG-TEP-TiO_2_ GPEs [61]. Reproduced from Ref. [61] with permission from Elsevier.

A flame-retardant GPE design can further increase the safety of LMBs. However, conventional LE flame retardants are not conducive to the electrochemical stability of batteries [89,90]. Fortunately, the introduction of a polymer with flame retardant properties into the polymer substrate can alleviate the problem of flame retardant and electrode incompatibility. Long et al. [91] prepared a P(AN-DEVP) multifunctional GPE (PAxDy) by phase separation. P(AN-DEVP) functional groups, the N and O atoms of phosphoric acid, and nitrile groups promote lithium ion transference by coupling and decoupling. Moreover, the phosphoric acid groups of DEVP undergo cyclization reactions with nitrile groups, making a pyknotic polymer network and preventing battery overheating. In addition, compared to LEs, PA1D1 has excellent interfacial compatibility with graphite anodes and good cyclic stability after being assembled into batteries with different cathodes (LiFePO_4_, NCM622), which are traits related to the stable growth of SEIs. Additionally, the synergistic action of flame-retardant polymers and nanoparticles in the construction of a fast ion transfer channel not only effectively improves the conductivity of GPEs, but also guarantees the safety of the battery. TEP used as flame retardant also reduces the crystallinity of the polymer substrate because of its small molecular properties (Figure 7a–c). Furthermore, the surface diffusion layer of TiO_2_ nanoparticles promotes lithium salt dissociation and lithium ion migration [61].

## 3. SEIs Formed by GPEs and Their Advantages

### 3.1. The Brief Overview of SEIs

In the 1970s, Dey et al. discovered that LMA surfaces have a protective crystal layer. In 1979, Peled et al. proposed the concept of the SEI, which serves as a barrier between the electrolyte and anode to improve the electrolyte’s dynamic stability. The SEI is similar to solid electrolytes, which are not electron conductors but ionic conductors [92,93,94]. Moreover, because ions and electrons continuously undergo electrochemical reactions at this interface, the composition and structure of the SEI have a profound effect on the battery cyclic stability. A brittle SEI can lead to undesirable side reactions that affect the battery performance [95,96]. When a lithium dendrite punctures the SEI, the uncontrolled consumption of the LE causes a decrease in Coulombic efficiency [97,98,99]. Therefore, a stable SEI is desirable.

#### 3.1.1. SEI Formation

**Figure 8:**
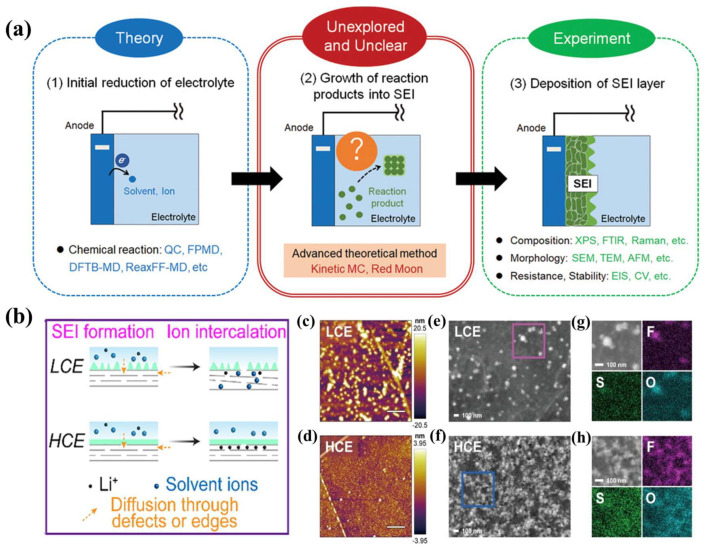
(**a**) Overview of experimental and theoretical analyses of the SEI formation process, consisting of three major steps [102]. Reproduced from Ref. [102] with permission from Wiley; (**b**) scheme of SEI formation and lithium ion intercalation in HCE and LCE; quasi in situ AFM of the SEI formed on HOPG electrodes under (**c**) LCE and (**d**) HCE conditions; SEM images and corresponding EDS spectra are shown in (**e**,**f**) for LCE conditions and (**g**,**h**) for HCE conditions [9]. Reproduced from Ref. [9] with permission from Elsevier.

The lowest unoccupied molecular orbital (LUMO) potential of the electrolyte is lower than that of the Fermi energy of the anode, resulting in a reduction reaction, which is a prerequisite for SEI formation. The SEI is composed of decomposition products of electrolytes [100,101]. The formation of a SEI involves three stages: (1) the reduction of electrolytes; (2) the formation of a SEI layer between the anode and electrolyte; (3) the deposition of a SEI (Figure 8a) [102]. In addition, the structure of electrolyte solvents has a direct effect on the structural stability of SEIs [103]. Lithium ethylene carbonate (LEC) generated by the reaction of DEC with the LMA has high dispersion in the electrolyte and cannot be used as a stable component of SEIs. In contrast, lithium ethylene decarbonate (LDEC) produced by EC reacting with the LMA has low dispersion in LEs. This is conducive to the formation of stable SEI layers. Moreover, the continuous lithium consumption of LEs is reduced, thus improving the battery’s Coulombic efficiency. Li et al. [9] investigated the effects of lithium content in electrolytes on SEI formation. Compared with low concentration electrolytes (LCEs), the high concentration electrolytes (HCEs) formed SEIs with more inorganic components, which are conducive to lithium ion transference (Figure 8b). Moreover, the HEC-derived SEI is especially flat and dense, which can effectively inhibit the intercalation of electrolyte solvent molecules (Figure 8c–h). Interestingly, an SEI layer is not formed between aqueous LiOH electrolytes and the anode during initial cycling [104]. Minakshi et al. studied the application of aqueous LiOH electrolytes in lithium batteries. An aqueous rechargeable lithium battery with MnO_2_ as a cathode and Zn as an anode has the advantages of low cost and environmental friendliness [105,106]. However, compared with organic electrolyte lithium batteries, there is still room for improvement in the energy density and electrochemical window of aqueous LiOH lithium batteries [107,108].

In addition, the effect of electrode material on SEIs should not be ignored. Electrode polarization and electronic properties are also important for SEI formation. For example, as graphene layers increase, the anode electronic structure is constantly changing. Electrons shift from vertical transfer to diffusion along the electrode layer, resulting in a slower SEI formation rate. Moreover, the Löwdin number of the lithium ion layer (L) and graphene layer (C) is significantly lower for symmetrical structures than for asymmetrical structures. In the morphologies of graphene anodes, a single layer of symmetrical graphene has the lowest Löwdin number. Therefore, a decrease in the Löwdin number indicates an increase in the reducibility of the electrode material, which is more conducive to SEI generation [109].

#### 3.1.2. Composition and Structure of SEIs

**Figure 9:**
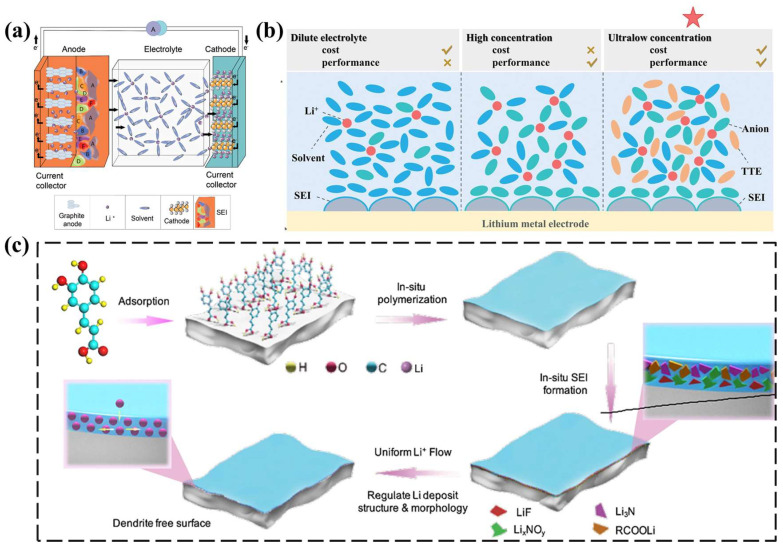
(**a**) Schematic of a conventional LIB detailing the SEI layer with a snapshot of the initial SEI structure formed on the graphite electrode interface [110]. Reproduced from Ref. [110] with permission from Wiley; (**b**) design models of different electrolytic liquid systems [113]. Reproduced from Ref. [113] with permission from Wiley; (**c**) schematic illustration of multifunctional SEI formation on the LMA [114]. Reproduced from Ref. [114] with permission from Nature Communications.

SEIs include organic regions next to the electrolyte (oligomers and lithium carbonate salts) and inorganic regions near the anode (Li_2_O, LiF, etc.) (Figure 9a) [110]. The outer organic region is a porous heterogeneous structure, where both lithium ions and solvent molecules can reach the interface. The inner inorganic region can transfer lithium ions [93,111]. However, if the SEI produced by electrolytes with the same properties has a similar composition, its structure is highly variable. Researchers found that even in two similarly composed LEs, LE-1(0.6M LiBF_4_ and 0.6M LiBOB, EC:EMC = 3:7, *v*/*v*) and LE-2 (1.2M LiDFOB, EC:EMC = 3:7, *v*/*v*), the cycle stability of electrolyte-2 is obviously better than that of electrolyte-1 [112]. This is mainly due to the formation of nanostructured LiF in the SEI of electrolyte-2. The capping ability of oxalic acid in LiDFOB can induce the uniform growth of nanostructured LiF. Nanostructured LiF in SEIs forms a diffusion field gradient on the surface of the LMA, which is beneficial to the cycle performance of the battery. In addition, the SEI formation rate is the key to achieving high Coulombic efficiency. HCE can induce dense lithium deposition. However, HCE is expensive and suffers from poor wettability. Chen et al. [113] used 1,1,2,2-tetrafluoroethyl-2,2,3,3-tetrafluoropropyl ether (TTE) to dilute electrolyte D5 (n_THF_:n_LiFSI_:n_TTE_ = 14:5:0, molar ratio) and prepared local high concentration electrolyte H1 (n_THF_:n_LiFSI_:n_TTE_ = 14:5:14, molar ratio). The solvation structure of lithium ions in H1 is consistent with that in D5, which maintains the high concentration effect of electrolyte (Figure 9b). Notably, the essence of an SEI is to reduce the thermodynamic properties of electrolytes and the kinetic properties of the LMA to reduce electrolyte consumption. Therefore, it is worth considering introducing additives that inhibit electrolyte decomposition to establish robust SEIs. Luo et al. [114] prepared a multifunctional SEI for dendritic anodes using catechol and acrylic groups as electrolyte additives (Figure 9c). Catechol reduces electrolyte consumption. The acrylic anion forms a uniform polymerization layer on the anode surface, which is beneficial to long-term cyclic stability.

### 3.2. The Relationship between GPEs and SEIs

**Figure 10:**
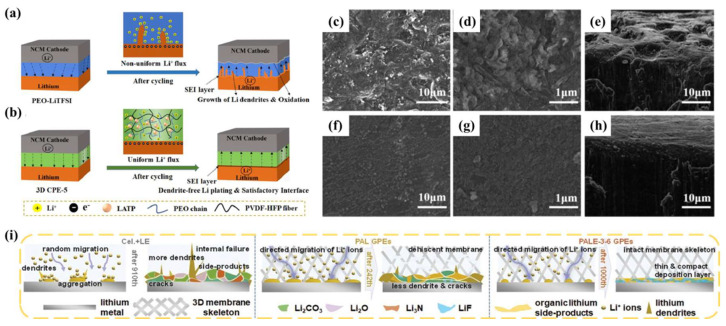
(**a**) Interfacial oxidation reaction and Li dendrites keep propagating (pure PEO electrolyte), and (**b**) introducing LATP powders and anti-oxidative PVDF–HFP fiber promotes the uniform deposition of lithium ions and improves the mechanical strength (3D CPE-5 separator) [122]. Reproduced from Ref. [122] with permission from Elsevier; SEM images of the lithium anodes after repeated stripping/deposition processes at a current density of 0.5 mA cm^−2^ using (**c**–**e**) PVDF GPE and (**f**–**h**) PVDF/PSPEG GPE [123]. Reproduced from Ref. [123] with permission from Elsevier; (**i**) the deposition mechanism of Li^+^ ions and a schematic diagram of the Li anode surface in batteries assembled with LE, PAL, and PALE-3-6 GPEs [124]. Reproduced from Ref. [124] with permission from Wiley.

The composition and structure of SEIs can be effectively regulated by introducing additives into the electrolyte. However, with the continuous charge-discharge cycle of the battery, the additives gradually fail, which is not favorable to the long-term stability of the lithium battery. An artificial polymer SEI layer constructed on the surface of the LMA can solve the problem of electrolyte additive failure [115,116,117]. Nonetheless, the electrochemical performance of the SEI layer formed by the electrolyte and electrode material during the initial charge and discharge is different from that of the artificial SEI layer constructed at the interface of the anode material [118]. The structure, composition, and mechanical properties of an artificial SEI can be precisely controlled to separate the anode and electrolyte. This design goal is the same as those of liquid electrolyte additives and polymer electrolytes. However, with the increase in battery charging and discharging times, the damaged artificial SEI cannot be repaired by the reduction products of the electrolyte [119,120,121]. As can be imagined, the artificial SEI is not a one-size-fits-all option for battery stability. The way to reduce the reactivity of LEs and LMAs without seriously sacrificing RT conductivity is to reduce the contact area between them. The GPE with a LE fixed on the polymer substrate meets this requirement. The GPE has good RT conductivity, the polymer substrate toughness can tolerate the volume expansion of the lithium anode, and the polymer substrate-fixed LE has anode wettability to reduce interfacial resistance. Under the combined action of the polymer base, lithium salt, and plasticizer or additive, the SEI stability of a GPE is higher than that of a pure LE. Yao et al. [122] used PVDF-HFP and ceramic-particle LATP to form a uniform SEI layer by in situ polymerization of a 3D network structure PEO-based composite polymer electrolyte. (Figure 10a,b). Shen et al. [123] prepared a GPE (PVDF/PSPEG GPE) composed of PVDF and an organic polysulfide polymer (PSPEG) for Li-S cells to form a stable SEI with Li_2_S/Li_2_S_2_ inorganic components and organic sulfide or polysulfide. It is delightful that Li|| PVDF/PSPEG GPE ||Li lithium batteries did not exhibit lithium dendrite formation after long cycling (Figure 10c–h). The functional group of the GPE polymer substrate is electronegative and can form a solvated structure of lithium ions, which is conducive to the uniform deposition of lithium ions. Chai et al. [124] prepared PALE GPEs using polyacrylonitrile/polylactic acid-block-ethylene glycol polymer (PALE) by adjusting the chain length and the cross-linked segments structure. The groups (-OH, -C-O-C-, -C=O, and -C≡N) on the polymer chain induce the migration of lithium ions along the polymer chain. Moreover, the cross-linked structure can buffer the volume change in the LMA (Figure 10i). Furthermore, Li|| PALE GPE-3-6 ||Li cell maintains stable cycling after 890 h at 0.5 mA g^−1^.

## 4. The Effect of GPE Performance on SEIs

The SEI is an indispensable component of the battery, and plays an important role in regulating lithium ion deposition [125,126]. Therefore, the generation of an SEI with uniform, appropriate thickness, good ionic conductivity, and good mechanical properties is necessary. GPEs can reduce the contact area between LEs and the LMA, and the functional groups on their polymer substrates contribute to the deposition of lithium ions, which is good for the formation of a robust SEI. In addition, this avoids the failure of an artificial SEI that cannot be repaired during the battery cycle and avoids the poor compatibility between the SPE and the LMA interface [127,128,129].

The GPE polymer substrate, lithium salts, plasticizers, additives, and even synthesis methods have significant effects on the composition and structure of SEIs. For example, polymer substrates have an effect on the dissociation and transference of lithium salts. Lithium salts (LiFSI, LiTFSI, LiPF_6_, etc.) control the inorganic composition of the SEI (LiF, Li_2_CO_3_, Li_2_O, etc.). Plasticizers (carbonates, ethers) direct the organic components of SEI (alkyl carbonates). Additives affect the composition and performance of the SEI. Therefore, it is necessary to study the influence of GPEs on SEIs.

### 4.1. Effect of GPE Preparation Method on SEIs

**Figure 11:**
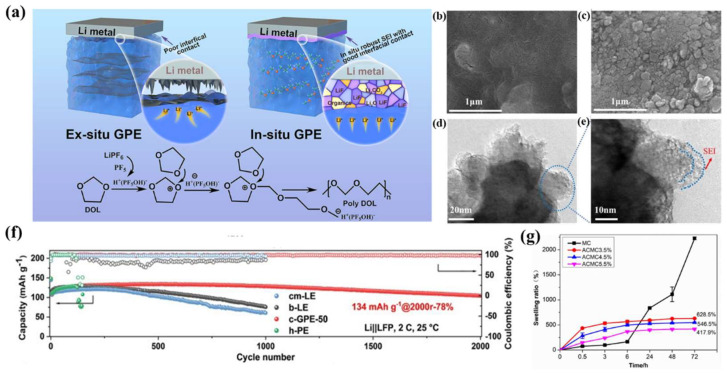
(**a**) Schematic illustration of the interfacial contact between the Li metal anode and the ex situ GPE or in situ GPE [134]. Reproduced from Ref. [134] with permission from American Chemical Society; (**b**) surface SEM images of the lithium anode of a Li−FEC||PPL−6.7%||Li−FEC battery after cycling for 200 h at 0.2 mA cm^−2^; (**c**) lithium anode of Li ||PPL−6.7%||Li battery after being tested for 120 h at 0.2 mA cm^−2^; (**d**,**e**) TEM of Li anodes of Li−FEC||PPL−6.7%||Li−FEC after cycling for 200 h [135]. Reproduced from Ref. [135] with permission from Elsevier; (**f**) cycling performances of Li||LFP batteries with cm−LE (blue), b−LE (black), c−GPE−50 (red), and h−PE (green) at 2 C [136]. Reproduced from Ref. [136] with permission from Elsevier; (**g**) the swelling property of film MC and ACMC3.5%, 4.5%, and 5.5% [140]. Reproduced from Ref. [140] with permission from Elsevier.

The problem of incomplete contact between the anode and the independently prepared polymer electrolyte leads to the impossibility of maximizing the capacity of the battery. Therefore, an in situ preparation method has been proposed [130]. In situ-prepared GPEs can solve the interface problem between the anode and polymer electrolyte. However, it is not enough to only solve the interface problem to improve battery performance [131,132,133]. Jiao et al. [134] used LiPF_6_ as an initiator to prepare a GPE in situ through ring-opening polymerization of DOL (Figure 11a). Moreover, a LiF-rich dense SEI was formed between a GPE and a lithium anode with the addition of FEC, which caused uniform deposition of lithium ions and improved cyclic stability. Wang et al. [135] prepared a sandwich structure GPE (PPL-6.7%) using in situ polymerization of PAN, trihydroxymethylpropyl trimethylacrylate (TMPTMA), and 1, 6-hexanediol diacrylate (HDDA). LMAs pretreated with FEC (Li-FEC) not only reduced interface impedance, but also formed a LiF-rich SEI layer. Li-FEC is still a smooth surface after Li-FEC || PPL-6.7% || Li-FEC cell cycles 100 times. Moreover, the existence of the SEI can stop the consumption of the electrolyte (Figure 11b–e). Zhu et al. [136] prepared a c-GPE through in situ cationic ring-opening polymerization of pentaerythritol glycidyl ether (PEE, a four-arm crosslinking agent) and DOL. When FEC is introduced, the compatibility of c-GPEs with high voltage cathodes is improved. Furthermore, compared to b-LE liquid electrolytes, c-GPE-50 forms LiF, B-O, and B-F SEI inner layers and polyether, LiN_x_O_y_ SEI outer layers. Consequently, Li ||c-GPE-50 || LFP cells achieved a lifespan of 2000 super long cycles, and their capacity retention rate was still 78% even at 2 C (Figure 11f).

In situ polymerization of GPEs has the main advantage of reducing interface resistance. However, there are still problems to be worked out: (1) volume change in the electrolyte before and after polymerization reactions; (2) controlling the degree of polymerization; (3) the influence of incomplete cells on battery performance; (4) appropriate initiation temperature, the reactivity of the initiator type to lithium metal, etc. [137,138,139]. In addition, the main problem of ex situ polymerized GPEs is that the mechanical properties decrease after activation in LEs. Therefore, it is imperative to improve the mechanical strength of the polymer substrate. The interaction between the polymer functional groups and the LE also needs to be enhanced. Yu et al. [140] prepared a GPE with good mechanical properties using allyl modified cellulose and methylcellulose triggered by UV irradiation. Methylcellulose has strong adsorption to LE (628.5%), improving the interface compatibility (Figure 11g). The introduction of allyl improves the mechanical strength and decreases the crystallinity of cellulose, thus increasing the ionic conductivity of the GPE (4.36 mS cm^−1^). Moreover, the GPE contains a large number of polar functional groups to enhance lithium salt dissociation and increase the lithium ion transference number to 0.902.

### 4.2. Effect of GPE Plasticizer Selection on SEIs

**Figure 12:**
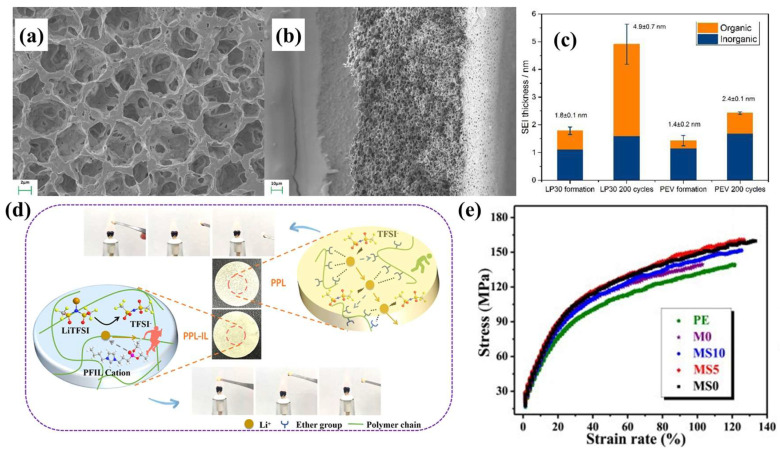
SEM image of a porous PVDF-HFP membrane: (**a**) surface; (**b**) cross-section and thickness change; (**c**) SEI formed in cells containing LP30-GPE and PEV-GPE upon cycling [11]. Reproduced from Ref. [11] with permission from Elsevier.; (**d**) schematic illustrations of the dual lithium-ion transport channels for the PPL-IL and flammability test [32]. Reproduced from Ref. [32] with permission from American Chemical Society; (**e**) mechanical stress-strain curves [34]. Reproduced from Ref. [34] with permission from Elsevier.

In order to promote the dissociation of lithium, the plasticizer must have a higher dielectric constant than lithium salt. Additionally, the plasticizer regulates the chemical composition of the SEI to form a stable interface between the electrolyte and the anode. High boiling point and high flash point plasticizers are beneficial to the safety performance of the battery [141,142,143]. An EC with a high dielectric constant is a common plasticizer used in GPEs. However, the high melting point of EC implies its use in combination with linear carbonate plasticizers. Jia et al. [11] prepared a porous GPE using PVDF-HFP, 1M LiPF_6_ in propylene carbonate (PC) +2wt% ethylene sulfite (ES) +2wt% vinylene carbonate (VC). The effect of the plasticizer on the SEI composition was studied (Figure 12a,b). Moreover, XPS analyzed the effects of different plasticizers on SEI composition before and after cycles. The SEI thickness of LP30-GPE without ES and VC increased significantly compared to that of the PEV-GPE without ES and VC after 200 cycles. The SEI organic outer layer thickness of the LP30-GPE without ES and VC increased significantly, which is not conducive to a stable battery cycle (Figure 12c).

Excessive decomposition caused by the high activity of organic carbonates reacting with the lithium anode will lead to uneven deposition/stripping of lithium. Notably, imidazole ionic liquid is a GPE plasticizer candidate due to its good conductivity and low viscosity. However, C2 of the imidazole ring is prone to protonation, resulting in poor cathode stability. Song et al. [32] introduced phosphate-functionalized imidazole ionic liquid (PFIL) into PEO and PAN polymers to prepare a GPE (PPL-IL). The phosphate groups not only improved the poor cathode reactivity, but also increased the GPE’s flame retardant performance. Moreover, the phosphorus oxide group reduced the interaction force between Li^+^ and TFSI^−^, which promoted the dissociation of lithium (Figure 12d). Surprisingly, PPL-IL can form a uniform and stable SEI layer rich in inorganic layers, inhibiting the growth of lithium dendrites. In addition, room temperature ionic liquids (RTILs) can decrease the crystallinity of polymer chains and increase the number of lithium ion transfers. However, excess IL can decrease the mechanical properties of GPEs [144,145]. Therefore, polymer ionic liquids (PIL) modified by IL functionalization have been extensively studied. Martinez-Ibañez et al. [146] used PIL as a polymer scaffold to prepare a high concentration bis(fluorosulfonyl)imide (FSI)-based ternary gel polymer electrolyte (FSI-TGPEs). The EIS test shows that Li || FSI-TGPE || Li symmetric cells have a lower interface resistance (830 Ω cm^−2^ at 25 °C, 19 Ω cm^−2^ at 70 °C) because the S-F bond of the FSI-anion formed a robust SEI. Furthermore, the synergistic actions of plasticizers and additives not only improve the mechanical properties of GPEs, but also generate stable SEIs. By introducing VC into an EMITSI plasticizer and adding nanoparticles (SiO_2_:Al_2_O_3_^1/4^ = 5:5) into poly(methyl methacrylate-acrylonitrile-ethyl acrylate) (P(MMA-AN-EA)), Li et al. [34] prepared a porous GPE with high mechanical properties (fracture strength 160 MPa) and high ionic conductivity (3.2 mS cm^−1^) (Figure 12e).

### 4.3. Effect of GPE Polymer Substrate on SEIs

**Figure 13:**
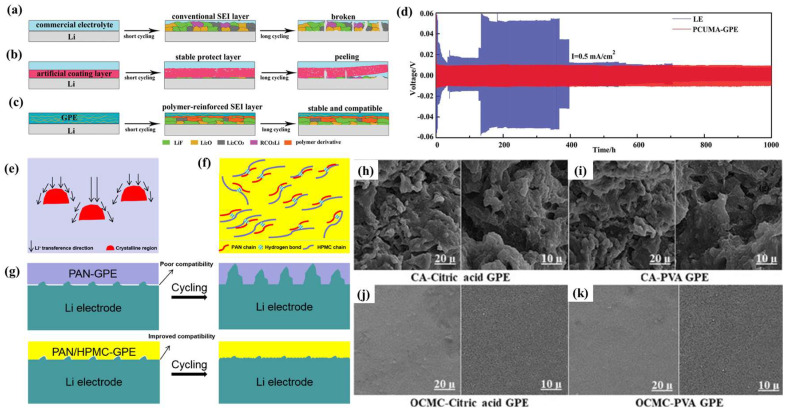
Schematic diagram of the evolution of SEI layers during cycling within the batteries: (**a**) commercially available LE-based LMBs, (**b**) commercial LE-based LMBs with artificial coating layers on the Li anode, and (**c**) GPE-assembled LMBs with in situ formed polymer-reinforced SEI layers on the Li anode; (**d**) Li plating/stripping experiments for symmetric Li/Li cells with PCUMA-GPE and the LE at a current density of 0.5 mA cm^−2^ [149] Reproduced from Ref. [149] with permission from Wiley-VCH.; (**e**) uneven Li^+^ transference direction in the PAN matrix; (**f**) a diagram of hydrogen bond interactions between PAN and HPMC; (**g**) a comparison of Li plating/stripping cycling with two types of GPEs [150]. Reproduced from Ref. [150] with permission from Elsevier; (**h**–**k**) FE-SEM of GPEs surfaces before cycling [37]. Reproduced from Ref. [37] with permission from Elsevier.

The polymer substrate includes crystalline regions that provide chemical stability and mechanical properties and non-crystalline regions that facilitate lithium ion transfer. The polymer properties include the dissociation of lithium salts, the fixation of LEs, and the formation of lithium ion complexes [147,148]. Hu et al. [149] prepared a PUCMA-GPE using cyclic carbonate urethane methacrylate (2-(((2-oxo-1, 3-dioxolan-4-yl) methoxy) carbonylamino) ethyl methacrylate) (CUMA). PCUMA is rich in rigid cyclic carbonate motifs with low LUMO levels, which are conducive to the formation of a strong SEI layer (Figure 13a–c). Moreover, Li || PUCMA-GPE ||Li cells display stable cycles for 1000 h at 0.5 mA cm^−2^ (Figure 13d). In addition, the interaction between different polymers is beneficial to the electrochemical performance of GPEs. PAN is widely used in GPE polymer substrates due to its excellent film forming properties and high ionic conductivity. However, PAN has the disadvantages of poor compatibility with LMAs and high crystallinity. Li et al. [150] prepared a GPE by mixing hydroxypropyl methyl cellulose (HPMC) with PAN. A large amount of -OH in HPMC can form hydrogen bonds with -CN in PAN, which can reduce the crystallinity of PAN. Furthermore, this interaction between hydrogen bonds improves the lithium ion transference and the interface compatibility with the anode (Figure 13e).

**Figure 14:**
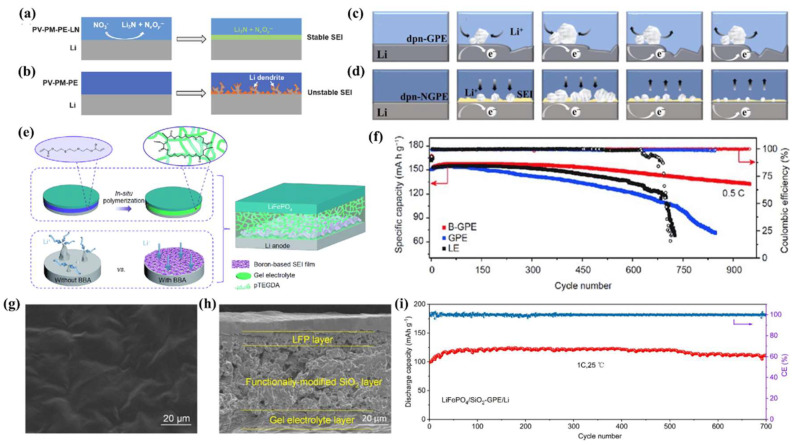
Schematic illustration of the electrochemical behavior of a LMA using a GPE (**a**) with LiNO_3_ and (**b**) without LiNO_3_ [72]. Reproduced from Ref. [72] with permission from Springer; schematic illustration of (**c**) the dpn-GPE/Li interface at OCP and (**d**) the dpn-NGPE/Li interface at OCP [159]. Reproduced from Ref. [159] with permission from Elsevier.; (**e**) schematic illustration of an assembly procedure and a microscopic model of a Li metal battery based on B-GPE; (**f**) cycling performance of Li||LiFePO_4_ batteries with different electrolytes at 0.5 C at 30 °C [160]. Reproduced from Ref. [160] with permission from Springer; SEM images of (**g**) surface and (**h**) cross-sectional morphology of the SiO_2_-GPE; (**i**) cycling performance of the LFP/SiO_2_-GPE/Li cell at 1 C [161]. Reproduced from Ref. [161] with permission from American Chemical Society.

Efficient and low-cost GPEs could reduce the manufacturing cost of polymer batteries. Cellulose is a suitable choice because it contains a large number of polar functional groups that can dissociate lithium salt. In addition, cellulose has the advantages of low cost, biodegradability, and good thermal stability. Hadad et al. [37] prepared a GPE using amorphous cellulose acetate (CA) and oxidized carboxymethyl cellulose (OCMC) as polymer substrates. Additionally, the effects of the long-chain crosslinking agent PVA and short-chain crosslinking agent citric acid on polymerization networks were compared (Figure 14h–k). Surprisingly, the GPE prepared with CA and OCMC showed excellent ionic conductivity, at ~10^−2^ S cm^−2^. The introduction of dilithium salts into the polymer substrate is conducive to the formation of a stable SEI. Lin et al. [151] prepared an SGPE with a separator (Celgard 2325) by blending PVDF-HFP with poly(2-hydroxyethyl methacrylate) (PHEMA) and introducing dilithium salts (LiTFSI and LiPF_6_). The introduction of PHEMA mainly reduces the crystallinity of PVDF-HFP and enhances the interfacial compatibility between the GPE and the LMA. There is no lithium dendrite formation after cycling the Li || SGPE || Li cell for 500 h. This suggests that the SGPE forms a stable SEI layer on the lithium metal surface.

### 4.4. Effect of GPE Additives on SEIs

The structure-activity relationships between the additives introduced in GPEs and SEIs effect the performance of the battery [152]. The additives need to have a LUMO level lower than lithium salt and the LE solvent [153,154,155]. Additives are generally divided into organic and inorganic additives. Inorganic additives include LiNO_3_, LiF, Li_2_Sn, boric acid, etc. LiNO_3_ can improve ionic conductivity and inhibit the S-shuttle effect in Li-S batteries. LiF can improve the battery cycle performance and CE [156,157]. Organic additives include FEC, VC, etc. Organic additives can adjust the components of the SEI and improve the SEI’s toughness [158]. In addition, the flexible layer and rigid layer of the SEI are regulated by the synergistic action of organic and inorganic additives.

LiNO_3_ has low solubility in carbonate LEs, so how to apply it to a carbonate LE is a problem that needs to be considered. Wang et al. [72] dissolved LiNO_3_ into polyether PEO solution to prepare a GPE (PV-PM-PE-LN). LiNO_3_ dissolved in PEO can generate a Li_3_N-containing SEI to improve ionic conductivity. Moreover, compared with a GPE (PV-PM-PE) without LiNO_3_, PV-PM-PE-LN can effectively regulate Li^0^ deposition (Figure 14a,b). It is well known that the structure and composition of SEIs change constantly during cycling. Therefore, real-time monitoring of the dynamic process can help to better understand the structure-activity relationship. In situ electrochemical atomic force microscopy (EC-AFM) can meet this detection requirement. Taking the structure-activity relationship between LiNO_3_ and SEIs as an example, EC-AFM can detect that the lithium of GPEs without LiNO_3_ is deposited unevenly and forms spherical nuclei. The volume of the spheres increases and more defects occur during cycling, which leads to the formation of lithium dendrites and a short circuit of the battery. In contrast, a LiNO_3_-containing GPE first forms a dense amorphous SEI layer, inducing the uniform deposition of lithium ions to form dense blocks. Furthermore, the stripping of lithium starts from the edge of the lithium block and then gradually involves the central position, which is conducive to interface stability and prevents the appearance of lithium dendrites (Figure 14c,d) [159].

The boron atom of boron acid is a Lewis acid. The PF_6_^−^ anion of LiPF_6_ is a Lewis base. B atoms and PF^6−^ cause a complex reaction which forms a B-containing SEI that inhibits dendrite formation. Han et al. [160] in situ polymerized TEGDA at 1 M LiPF_6_ (EC/DMC/DEC = 1:1:1, *v*/*v*/*v*) using azodihetonitrile (ABVN) heat initiation, then introduced n-butyl boric acid (BBA) into the GPE precursor solution to form a B-GPE (Figure 14e). Compared with the LFP || GPE || Li cells, the LFP || B-GPE || Li cell capacity retention rate is still 87.7% after 950 cycles at 0.5C. This is due to the fact that BBA forms a stable SEI to promote the battery circulation performance (Figure 14f). The doping mode of additives directly influences battery performance. SiO_2_ is the most commonly used ceramic additive, and its main role is to degrade the crystallinity of polymer substrates and thereby improve ionic conductivity. Uneven dispersion occurs when SiO_2_ is introduced directly into polymer precursors. Noteworthily, the surface modification of SiO_2_ can solve this problem. Yang et al. [161] coated SiO_2_ modified by 3-isocyana-topropyl-triethoxysilane (IPTS) and DOL on the LFP cathode through in situ preparation of SiO_2_-GPE (Figure 14g,h). The modified SiO_2_ effectively reduces the interfacial resistance and forms a stable SEI. The retention rate of the LFP|| SiO_2_-GPE||Li cell is 88.42% after 700 cycles at 1 C (Figure 14i).

## 5. Conclusions and Outlook

The high reactivity between LEs and LMAs leads to the formation of fragile SEIs and lithium dendrites, causing low Coulombic efficiency and serious safety problems for LMBs. GPEs possess the advantages of both SPEs and LEs, reducing the contact surface between the LE and the LMA while preserving LE-like ionic conductivity. Therefore, GPEs have a potential advantage in the practical application of LMBs. This review focuses on the effects of GPE composition and preparation methods on SEIs. Some existing problems and their remedies are summarized. (1) The initiator is involved in the formation of SEIs by in situ polymerization. At present, the most commonly used method is to use suitable lithium salt as initiator to induce polymerization. (2) The introduction of ceramic nanoparticles, MOF, and glass fiber, etc., can improve the mechanical properties of GPEs after plasticization. Furthermore, the introduction of these additives can promote the dissociation of lithium salt and reduce the concentration polarization. This is conducive to the formation of stable SEI layers. (3) High crystallinity of polymer substrates is not conducive to lithium ion transference. The introduction of nanoparticles (Al_2_O_3_, TiO_2_, GO nanosheets, vermiculite sheets, MXene, etc.) can reduce the crystallinity of the polymer substrate. Furthermore, high lithium ion transference numbers can effectively avoid concentration polarization and inhibit the formation of dead reason. It is beneficial to improve the stability of the SEI layer. (4) Carbonates are commonly used as plasticizers for GPEs. However, linear carbonate plasticizers have the defects of low flash points and dielectric constants, although their low viscosity is conducive to lithium dissociation. Cyclic carbonate plasticizers are the opposite. Therefore, the combination of circular carbonate and linear carbonate plasticizers can improve the electrochemical performance of batteries.

Although GPEs have made great progress in inhibiting the formation of lithium dendrites, several challenges remain. (1) Increasing the LE absorption rate of GPEs is conducive to improving the ionic conductivity of GPEs. However, a high LE absorption rate will lead to deteriorated stability of the SEI, causing safety problems. (2) How to effectively reduce the transference path of lithium ions in GPEs and improve the power density of LMBs should be considered. (3) GPEs improve interfacial compatibility with lithium anodes. However, the interfacial resistance of GPEs with different lithium battery cathodes needs to be investigated. (4) Under the initiative of green chemistry, it is imperative to choose natural polymer materials as GPE polymer substrates. (5) Understanding of SEIs is still limited. It is necessary to use real-time monitoring technology to characterize the composition and structural changes of SEIs. Furthermore, the components of SEIs are constantly changing. The existing calculation model is periodic. It cannot represent the SEIs formed during the whole cycling.

In summary, GPEs have practical applications in replacing LEs in LMBs, but they also presents serious challenges. The dynamic formation process and action mechanism of SEIs formed by GPEs need further study. In addition, GPEs are currently only available in the laboratory. Industrial production of GPEs is low. Additionally, the disadvantages of high price, low compatibility between different lithium batteries, and lack of a fixed model leads to poor market sustainability. Therefore, a great deal of energy needs to be invested in scientific research. We believe that GPEs can be widely used in the LMB market in the near future.

## Data Availability

No new data were created.

## Abbreviations

EMC	ethyl methyl carbonate
DMC	dimethyl carbonate
DEC	diethyl carbonate
PC	propylene carbonate
EC	ethylene carbonate
DME	1,2-dimethoxyethane
TEGDME	Tetraethylene glycol dimethyl ether
DOL	dioxolane
THF	tetrahydrofuran
FEC	fluoroethylene carbonate
LiDFOB	Lithium Difluoro(oxalato)borate
LATP	Li_1.3_Al_0.3_Ti_1.7_(PO_4_)_3_
